# Functional Analysis of Naturally Integrated *Rol* Genes in Sweet Potato via CRISPR/Cas9 Genome Editing

**DOI:** 10.3390/plants14243708

**Published:** 2025-12-05

**Authors:** Yury Shkryl, Yulia Yaroshenko, Valeria Grigorchuk, Victor Bulgakov, Yulia Yugay

**Affiliations:** Federal Scientific Center of the East Asia Terrestrial Biodiversity of the Far East Branch of Russian Academy of Sciences, 159 Stoletija Str., 690022 Vladivostok, Russia

**Keywords:** *Ipomoea batatas*, cT-DNA, *Agrobacterium rhizogenes*, callus culture, *rol* genes, secondary metabolism, genome editing

## Abstract

Sweet potato (*Ipomoea batatas*) is a globally important crop and one of a growing number of plants recognized as naturally transgenic, harboring *Agrobacterium*-derived T-DNA genes whose functions remain largely uncharacterized. In this proof-of-concept study, we applied CRISPR/Cas9 technology to generate targeted knockouts of the *Ib-rolB/C* and *Ib-rolD-like* genes located within the sweet potato cellular T-DNA2 (*Ib*T-DNA2) region. Mutations were introduced into sweet potato callus cultures using an optimized genome editing protocol, with most edits consisting of single-nucleotide insertions. Knockout of *Ib-rolB/C* did not affect callus growth but significantly reduced levels of chlorogenic acid derivatives. Validation *in planta* using transient expression in *I. batatas* leaves confirmed the suppressive effect of *Ib-rolB/C* disruption on polyphenol content. In contrast, *Ib-rolD-like* knockout lines showed reduced biomass accumulation and downregulation of cell cycle–related genes, but did not display significant changes in metabolite content in either callus cultures or leaf tissues. These findings suggest that *Ib-rolB/C* and *Ib-rolD-like* may differentially contribute to growth and secondary metabolism in sweet potato.

## 1. Introduction

Horizontal gene transfer (HGT) is a fascinating biological phenomenon involving the exchange of genetic material between organisms outside of traditional parent-to-offspring inheritance. Although widely recognized in prokaryotes, HGT has also played a pivotal role in eukaryotic evolution, including that of mammals and plants, where it contributes to increased genetic diversity and species evolution [1]. One particularly striking example in plants is the transfer of T-DNA from *Agrobacterium* species to plant genomes. This process occurs widely in nature, particularly at wound sites in dicotyledonous plants [2]. It typically causes neoplastic cell transformation, though the effects are usually limited to the affected individual’s development. However, it can sometimes lead to the fixation of integrated, or cellular (c)T-DNA, leaving a lasting imprint on the genomes of several plant species, which, as a result, become naturally transgenic [3]. Moreover, individual integration events can be repeated in representatives of naturally transgenic species, resulting in the appearance of multiple copies of cT-DNA from different species of *Agrobacterium* [4]. Beyond challenging traditional views of plant evolution and domestication, these events raise intriguing questions about the functional significance of foreign DNA, particularly its potential contributions to plant adaptation and phenotypic diversity.

Sweet potato (*Ipomoea batatas*), renowned as one of the world’s most important crops, holds immense nutritional and economic value, serving as a vital source of energy, vitamins, and minerals for millions worldwide [5]. Beyond its agronomic importance, sweet potato is unique among crops due to the presence of naturally integrated transgenes in its genome [6]. The sweet potato genome contains two distinct cellular T-DNA regions, designated *Ib*T-DNA1 and *Ib*T-DNA2. Notably, *Ib*T-DNA2 harbors a gene homologous to *A. rhizogenes rolB* and *rolC* (termed *Ib-rolB/C*), as well as an *Ib-rolD-like* gene, which encodes a protein with a characteristic NADB Rossmann-fold domain and ornithine cyclodeaminase activity, functionally analogous to the canonical *rolD* enzyme from wild-type *A. rhizogenes* A4 [7,8]. These transgenic loci are not unique to a single variety: the structure of *Ib*T-DNA regions, including *rol*-like genes, is conserved across several cultivated *Ipomoea* batatas genotypes. *Ib*T-DNA1 has been identified in all 291 examined sweet potato cultivars but not in closely related wild species, whereas *Ib*T-DNA2 was found in 45 out of 217 genotypes, including both cultivated and wild *Ipomoea* species [7]. Moreover, the recently published phased chromosome-level genome assembly of the ‘Tanzania’ cultivar [9] revealed the same *rol*-like genes and other open reading frames (ORFs) within the *Ib*T-DNA regions as had been previously reported in the ‘Huachano’ and ‘Taizhong’ cultivars [6,7]. Understanding the functions of these natural transgenes could yield valuable insights into their contribution to plant physiology, development, and adaptation.

The *rol* genes from *A. rhizogenes* are known to influence various plant processes, including modulation of defense mechanisms and activation of secondary metabolism, with the strongest effects observed for the *rolB* and *rolC* genes [10]. In addition, these genes affect cell growth in opposite ways: *rolB* inhibits growth, potentially inducing necrosis when strongly expressed, whereas *rolC* mitigates the negative effects of *rolB* and can independently stimulate cell growth [11]. Regarding the functional features of the *rolD* gene, its contribution to secondary metabolism appears to be minor [10], whereas its role in plant growth and development has been linked to proline production and associated downstream effects [12]. Additionally, *rolD* has been shown to enhance plant defense responses, as indicated by the upregulation of pathogenesis-related genes such as *PR-1* and increased resistance to toxins produced by pathogens like *Fusarium oxysporum* [13].

Evidence suggests that naturally integrated *rol*-like genes in *I. batatas*, such as *Ib-rolB/C* and *Ib-rolD-like*, may function in signaling pathways regulating plant stress responses and metabolism. The *Ib-rolB/C* gene shows age- and stress-induced expression and is strongly upregulated during leaf senescence and upon treatment with salicylic acid [14,15]. Functional studies in *Arabidopsis* indicate that *Ib-rolB/C* influences hormone signaling, promotes early flowering and leaf senescence, but does not affect root development. Moreover, its overexpression in *I. batatas* callus under a viral promoter has been shown to significantly increase the accumulation of chlorogenic acid derivatives, compounds associated with antioxidant activity and defense responses [16]. Another notable feature of sweet potato cT-DNA is the presence of an *Ib-rolD-like* gene, which encodes a protein with ornithine cyclodeaminase activity involved in proline biosynthesis [8]. Its expression is strongly induced by methyl jasmonate, and its heterologous expression in *Arabidopsis* suggests a role in growth regulation and reproductive development under stress conditions. These functions may contribute to enhanced metabolic efficiency, stress tolerance, or adaptive potential in *I. batatas* [17].

CRISPR/Cas9 genome editing technology has revolutionized plant genetics by enabling precise and targeted manipulation of genes [18,19]. While its widespread use in enhancing crop traits and elucidating gene functions in domesticated plants is well-documented [20], its application to natural transgenes remains a relatively unexplored frontier. Leveraging CRISPR/Cas9 to study the roles of naturally integrated transgenes, such as those in *I. batatas*, introduces an innovative approach to unraveling fundamental questions in plant genomics and evolution.

Plant cell cultures present a particularly promising platform for CRISPR/Cas9-mediated research [21]. These cultures offer a controlled and scalable environment for investigating gene function, especially in cases where whole-plant studies may be impractical or resource-intensive [22]. Furthermore, plant cell cultures have historically served as the primary model systems for functional studies of *Agrobacterium*-derived *rol* genes, owing to their well-established roles in regulating both secondary metabolite biosynthesis and growth-related processes under in vitro conditions [10,23,24].

The objective of this study was to elucidate the functional roles of the *Ib-rolB/C* and *Ib-rolD-like* genes in sweet potato using CRISPR/Cas9-mediated targeted mutagenesis. We hypothesized that, by analogy with their *Agrobacterium* homologs, these natural transgenes may contribute to the regulation of secondary metabolism and biomass accumulation. To test this hypothesis, we generated knockout lines and assessed their phenotypic, biochemical, and transcriptional profiles. This approach provides a framework for dissecting the functions of naturally integrated transgenes in a controlled in vitro system, which can serve as a basis for future studies in whole-plant models.

## 2. Results

### 2.1. Evaluation of Mutation Efficiency in CRISPR/Cas9-Transformed Callus Cultures of Ipomoea batatas

To optimize CRISPR/Cas9-based genome editing in *Ipomoea batatas* callus cultures, we assessed the influence of three key parameters: the *Agrobacterium* strain, bacterial suspension density, and callus age on mutation efficiency. All experiments employed binary vectors carrying the pcoCas9 gene and guide RNAs targeting *Ib-rolB/C* or *Ib-rolD-like*. To assess mutation frequency, transformed calli were cultured on selective medium for two weeks following transformation, after which genomic DNA was isolated for analysis. Mutation frequencies were determined by restriction enzyme digestion followed by quantification of band intensities using gel image analysis.

**Figure 1:**
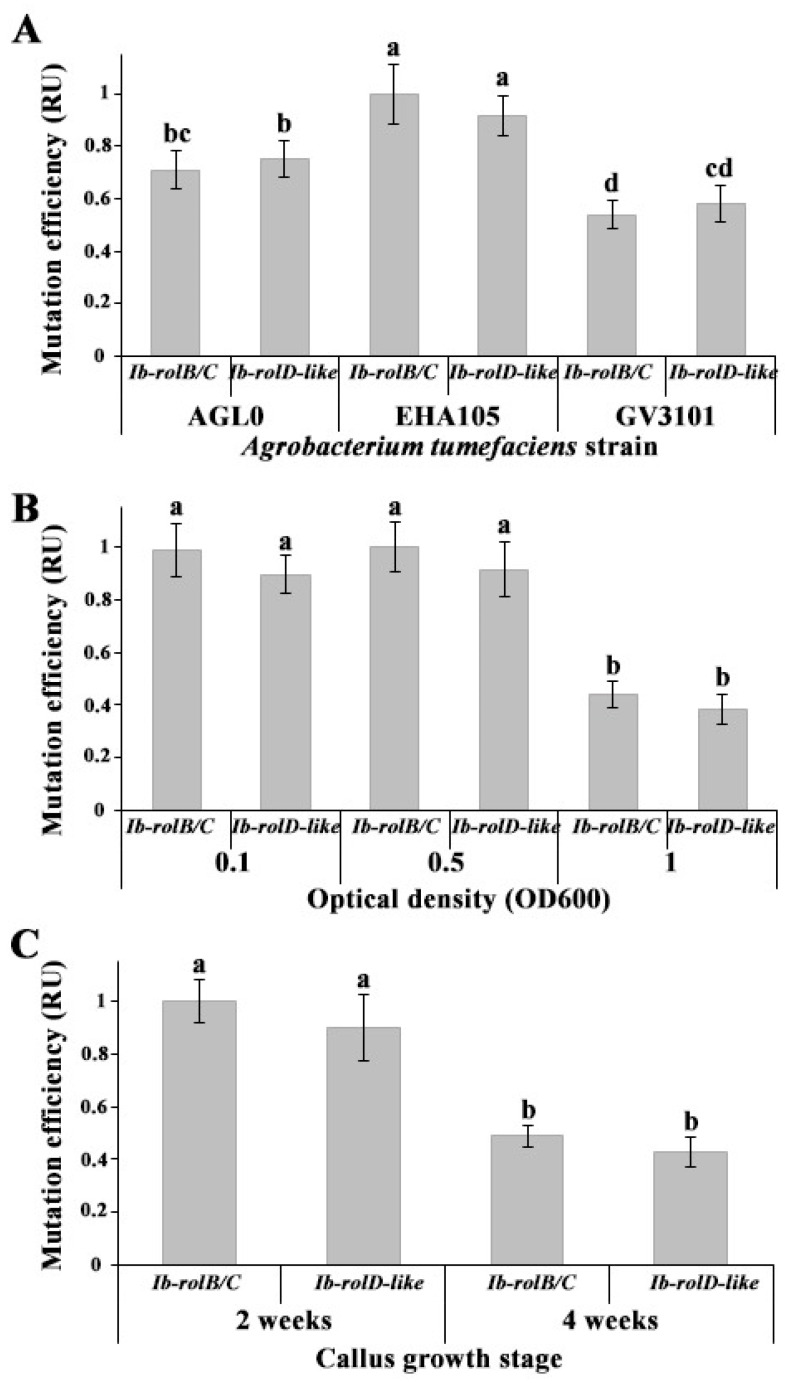
Effects of transformation parameters on CRISPR/Cas9-mediated mutation efficiency in *Ib-rolB/C* and *Ib-rolD-like* genes. (**A**) Comparison of *Agrobacterium tumefaciens* strains (EHA105, AGL1, GV3101). (**B**) Effect of bacterial suspension density (OD_600_ = 0.1, 0.5, 1.0). (**C**) Influence of callus age (2 or 4 weeks) on transformation outcomes. Mutation rates were determined two weeks after transformation. Target genomic regions were amplified by PCR, digested with restriction enzymes, and resolved by agarose gel electrophoresis. Band intensities were quantified using gel image analysis, and efficiency values are expressed in relative units (RUs), normalized to the highest value within each comparison. Values are presented as means ± standard errors. Different letters indicate statistically significant differences in means (*p* < 0.05), Fisher’s LSD.

Transformation efficiency is known to vary markedly among *Agrobacterium* strains and depends strongly on the host plant species [25]. In this study, three commonly used strains, AGL0, EHA105, and GV3101, were tested under identical conditions. Among them, strain EHA105 consistently produced the highest mutation rates in both target genes (Figure 1A). AGL0 showed moderate efficiency, whereas GV3101 demonstrated the lowest transformation capacity.

To determine the optimal bacterial density, we compared OD_600_ values of 0.1, 0.5, and 1.0. The mutation efficiencies obtained with OD_600_ = 0.1 and 0.5 were comparable and statistically indistinguishable (Figure 1B). However, higher bacterial concentrations led to increased tissue necrosis and reduced callus viability, thereby complicating the selection of mutant lines. These effects are consistent with previous reports in other plant species [26,27]. At OD_600_ = 1.0, a marked decrease in mutation efficiency was observed, indicating that excessive bacterial load adversely affects transformation outcomes. Based on these results, OD_600_ = 0.1 was selected as the optimal condition for further experiments.

The physiological condition of the plant material also plays an important role in transformation success [28]. To evaluate the effect of callus developmental stage, *I. batatas* calli aged two or four weeks were subjected to transformation under otherwise identical conditions. As shown in Figure 1C, the younger, two-week-old calli exhibited significantly higher mutation frequencies than four-week-old tissues. The lower transformation efficiency observed in older calli may be attributed to age-dependent physiological and cellular changes that limit the efficiency of Agrobacterium-mediated gene transfer. Transformation efficiency is known to critically depend on the “competency of target tissues,” which tends to decline with plant age [29]. Mature cells typically display increased chromatin condensation and heterochromatin formation, which may restrict access to T-DNA integration sites [30,31]. Additionally, transformation success is closely linked to cell cycle phase, with cells in the S and G2 phases showing higher susceptibility to T-DNA integration [32]. Older tissues may also mount stronger hypersensitive responses, leading to localized necrosis that interferes with efficient T-DNA delivery [33,34]. Although these mechanisms were not directly examined in our study, they offer plausible explanations for the observed decline in transformation efficiency with increasing callus age.

### 2.2. Cas9/gRNA-Mediated Genome Editing in Sweet Potato Callus Culture

**Figure 2:**
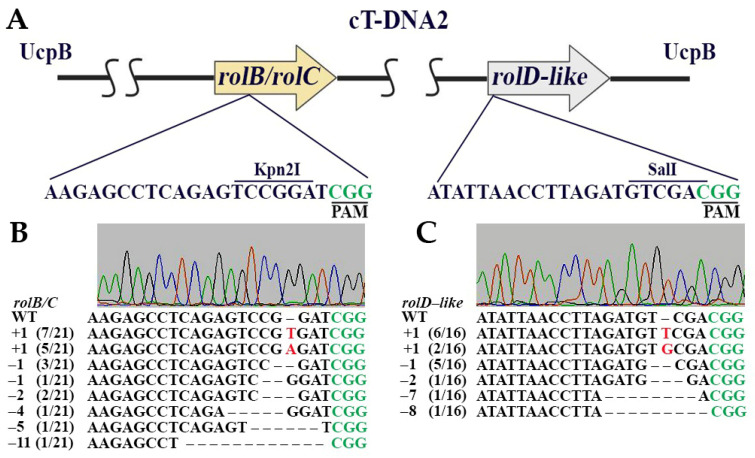
Targeted genome engineering in sweet potato leaves using the Cas9/gRNA system. (**A**) Schematic illustration of the gRNA target sites within the *Ib-rolB/C* and *Ib-rolD-like* genes. The detailed sequences below show the target sites for gRNA1 and gRNA2, with the protospacer sequences in black and the protospacer adjacent motifs (PAMs) in green. The restriction enzyme cutting sites (*Kpn*2I for *Ib-rolB/C* and *Sal*I for *Ib-rolD-like*) are indicated. Mutations induced by Cas9/sgRNA in the *Ib-rolB/C* (**B**) and *Ib-rolD-like* (**C**) genes in sweet potato, aligned with the corresponding wild-type (WT) sequences. The PAM sites are shown in green, and the mutations are shown in red. Insertions (+) and deletions (−) are indicated relative to the WT sequence. The frequency of each mutation is indicated in brackets.

Using *A. tumefaciens*-mediated transformation, we delivered Cas9/gRNA constructs into *I. batatas* callus cultures. Transgenic lines were selected based on resistance to the selective antibiotic kanamycin and the presence of the *nptII* gene, confirmed by PCR analysis. Three independent transgenic lines were obtained for each variant of Cas9/gRNA constructs. Callus lines in which the *Ib-rolB/C* gene was targeted for mutagenesis were designated “BC”, while lines in which the *Ib-rolD-like* gene was edited were designated “DL”. Mutations in the targeted genes were analyzed using total DNA isolated from the transgenic callus lines. Fragments resistant to restriction enzyme digestion were isolated from agarose gels, cloned, and sequenced. Several mutant alleles were identified for each target gene (Figure 2). For *Ib-rolB/C*, the most frequent mutations were single-nucleotide insertions of T and A, accounting for approximately 57% of all mutations. Additionally, deletions ranging from 1 to 11 bp were observed. In *Ib-rolD-like*, the predominant mutation types were insertions of the nucleotide T and single-base deletions, which together comprised 75% of the total mutations. In both genes, insertions and deletions occurred at various positions near the Cas9 cleavage site, leading to frameshift mutations and premature stop codons that resulted in loss-of-function alleles.

### 2.3. Impact of Ib-rolB/C and Ib-rolD-like Gene Mutations on the Growth of Ipomoea batatas Callus Cultures

**Figure 3:**
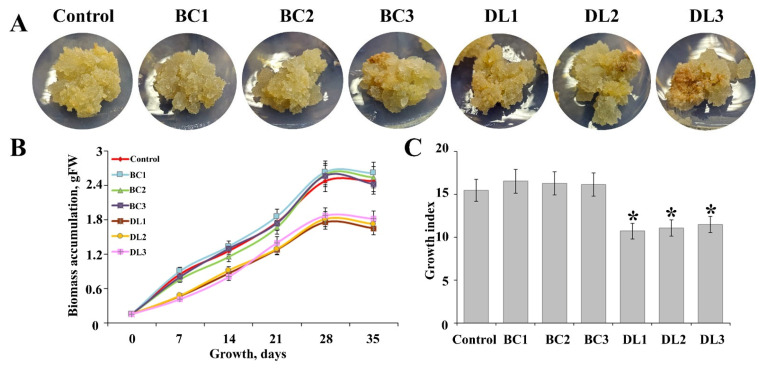
Growth characteristics of control and transgenic *Ipomoea batatas* callus lines with CRISPR/Cas9-induced mutations in *Ib-rolB/C* and *Ib-rolD-like* genes. (**A**) Representative images of control and transgenic calli after 35 days of cultivation. (**B**) Growth curves showing biomass accumulation (fresh weight, FW) over 35 days of cultivation. (**C**) Growth indices (ratio of biomass at day 35 to biomass at day 0) of control and transgenic calli. Data are presented as mean ± SE, with asterisks indicating significant differences from the control (*p* < 0.05).

The growth dynamics of control and transgenic *I. batatas* calli edited for *Ib-rolB/C* and *Ib-rolD-like* genes were assessed over a 35-day cultivation period. The appearance, biomass accumulation, and growth indices of these calluses are summarized in Figure 3.

The morphological analysis (Figure 3A) revealed distinct differences between the control and transgenic calli. The control callus line exhibited dense and compact growth with a uniform texture. Similarly, *Ib-rolB/C*-edited lines showed comparable morphology, suggesting that these mutations did not significantly affect cell structure. In contrast, *Ib-rolD-like* mutants displayed a looser texture and areas of discoloration, indicating possible disruptions in cellular organization or metabolism.

Biomass accumulation was monitored over time, and growth curves were plotted (Figure 3B). The fresh weight (FW) of the control and *Ib-rolB/C* mutants increased steadily, with no significant differences in growth kinetics. However, the growth rates of the *Ib-rolD-like* mutants were noticeably slower, particularly after 14 days of cultivation, resulting in significantly reduced biomass accumulation by day 35. This indicates that mutations in the *Ib-rolD-like* gene may interfere with growth-promoting pathways in sweet potato calli.

To quantify these differences, growth indices were calculated as the ratio of biomass at day 35 to biomass at day 0 (Figure 3C). The growth indices of *Ib-rolB/C* mutants were comparable to those of the control, confirming that mutations did not negatively impact growth. In contrast, the *Ib-rolD-like* mutants exhibited significantly lower growth indices, which were approximately 27% lower than those of the control culture. These findings suggest that the *Ib-rolD-like* gene plays a critical role in regulating cell growth, and its disruption has a more pronounced effect compared to *Ib-rolB/C*.

### 2.4. Effect of Ib-rolB/C and Ib-rolD-like Gene Mutations on Secondary Metabolite Levels in Ipomoea batatas Callus Cultures

The impact of CRISPR/Cas9-induced mutations in the *Ib-rolB/C* and *Ib-rolD-like* genes on the accumulation of secondary metabolites was analyzed in sweet potato callus lines BC1–3 and DL1–3, respectively. Several quinic acid derivatives were identified, including chlorogenic acid (CGA) and six diacyl derivatives of quinic acid, with 3,5-dicaffeoylquinic acid (3,5-diCQA) being the predominant compound across all samples. Other detected metabolites included *3,4-di-O-caffeoylquinic acid* (3,4-diCQA), *4,5-di-O-caffeoylquinic acid* (4,5-diCQA), *3-O-caffeoyl-5-O-coumaroylquinic acid* (3C-5CoQA), *3-O-feruloyl-5-O-caffeoylquinic acid* (3F-5CQA), and *3-O-caffeoyl-5-O-feruloylquinic acid* (3C-5FQA). Notably, CQAs represented the predominant class of secondary metabolites detected in *I. batatas* callus under our experimental conditions (Supplementary Figure S1).

The results (Table 1) demonstrated a significant reduction in the levels of total CQAs in *Ib-rolB/C* mutant callus lines (BC1–3) compared to the wild-type (WT) callus. In particular, the content of 3,5-diCQA was approximately 2.5 times lower in the BC lines compared to WT. Similarly, other CQAs, such as 4,5-diCQA, 3C-5CoQA, 3F-5CQA, and 3C-5FQA showed significant reductions in BC lines. In contrast, while total CQAs and individual metabolites showed a downward trend in *Ib-rolD-like* mutant cultures (DL1–3) compared to WT, the observed reductions were not statistically significant.

Overall, these findings suggest that mutations in *Ib-rolB/C* have a pronounced impact on the biosynthesis of CQAs, whereas the effect of *Ib-rolD-like* mutations on secondary metabolite levels is relatively minor.

### 2.5. Expression Profiles of Key Biosynthetic and Growth-Related Genes in Mutant Ipomoea batatas Calli

To investigate the molecular mechanisms underlying the effects of mutations in the natural *Ib-rolB/C* and *Ib-rolD-like* transgenes, we analyzed the expression of key genes involved in secondary metabolism and growth in mutant sweet potato callus cultures.

**Figure 4:**
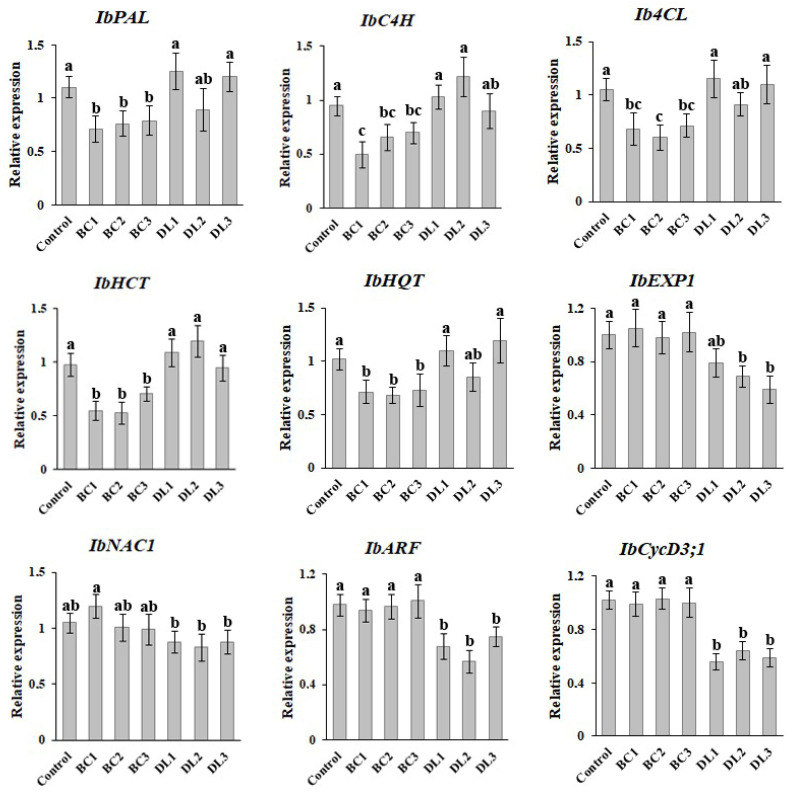
Expression analysis of genes involved in secondary metabolism (*IbPAL*, *IbC4H*, *Ib4Cl*, *IbHCT*, *IbHQT*, and *IbEXP1*) and growth (*IbNAC1*, *IbARF*, and *IbCycD3;1*) in control and transgenic *Ipomoea batatas* callus lines with CRISPR/Cas9-induced mutations in *Ib-rolB/C* and *Ib-rolD-like* genes. Values are presented as means ± standard errors. Different letters indicate statistically significant differences in means (*p* < 0.05), Fisher’s LSD.

The impact of CRISPR/Cas9-induced mutations in *Ib-rolB/C* (BC1–3) and *Ib-rolD-like* (DL1–3) genes on the expression of key biosynthetic genes involved in CQAs pathways was analyzed. In callus cultures with *Ib-rolB/C* mutations (BC1–3), the expression levels of *IbPAL*, *IbC4H*, *Ib4CL*, *IbHCT*, and *IbHQT* were significantly reduced compared to the control callus line (Figure 4). The decrease in *IbPAL* expression was most pronounced, with BC lines showing an average reduction of approximately 40%. In contrast, although slight variations in expression levels were observed in callus cultures with *Ib-rolD-like* mutations (DL1–3), the differences in the expression levels were not statistically significant compared to the control.

The influence of *Ib-rolB/C* and *Ib-rolD-like* mutations on the expression of key cell growth-related genes was also analyzed. In *Ib-rolB/C* mutant callus lines (BC1–3), the expression levels of *IbEXP1*, *IbNAC1*, *IbARF*, and *IbCycD3;1* genes showed minor fluctuations compared to the control calli (Figure 4). In contrast, *Ib-rolD-like* mutant lines (DL1–3) displayed a statistically significant reduction in the expression of *IbCycD3;1*, a key regulator of the G1-to-S transition in the cell cycle. Additionally, moderate but significant decreases in *IbARF* and *IbEXP1* expression were observed, suggesting that *Ib-rolD-like* mutations may impair auxin signaling and cell wall dynamics, contributing to reduced cell proliferation. The expression of *IbNAC1* remained largely unchanged in the DL lines, indicating that it is not strongly influenced by *Ib-rolD-like* gene mutations.

### 2.6. Effect of Ib-rolB/C and Ib-rolD-like Gene Mutations on Phenolic Compound Accumulation in Planta

**Figure 5:**
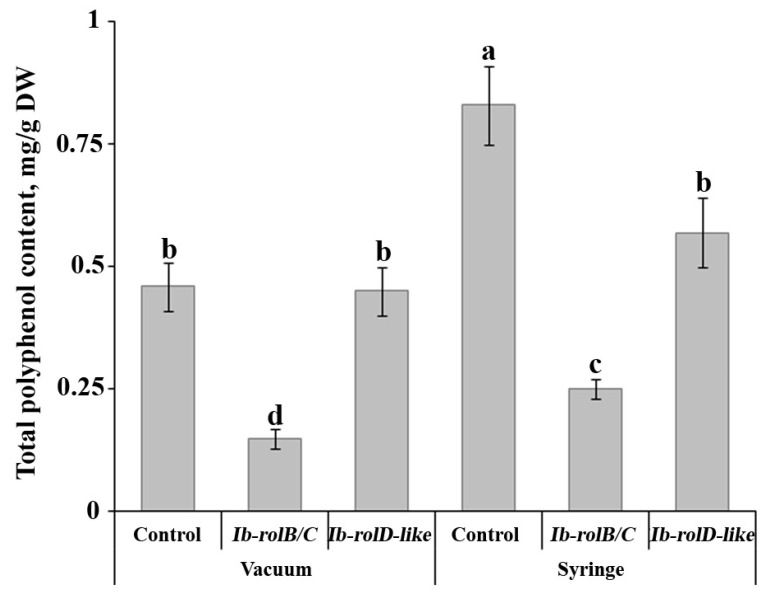
Effect of CRISPR/Cas9-induced mutations in the *Ib-rolB/C* and *Ib-rolD-like* genes on the accumulation of phenolic compounds in *Ipomoea batatas* leaves. Values are presented as means ± standard errors. Different letters indicate statistically significant differences in means (*p* < 0.05), Fisher’s LSD.

To evaluate the impact of CRISPR/Cas9-induced mutations in the *Ib-rolB/C* and *Ib-rolD-like* genes on the accumulation of secondary metabolites in intact sweet potato tissues, transient transformation of leaves was performed using gene-specific constructs. As a control, leaves were infiltrated with *Agrobacterium* carrying the empty vector. Quantitative analysis of total phenolic content revealed a significant reduction in leaves with mutations in the *Ib-rolB/C* gene compared to the control group (Figure 5). In contrast, leaves transformed with constructs targeting *Ib-rolD-like* did not exhibit statistically significant changes in phenolic metabolite levels. In all experimental variants, syringe agroinfiltration led to a noticeable increase in phenolic compound accumulation relative to untreated controls. This effect is likely attributable to the activation of defense-related metabolic pathways in response to mechanical wounding during infiltration.

## 3. Discussion

Sweet potato (*Ipomoea batatas*) is the sixth most important food crop globally, with annual production exceeding 105 million metric tons [35]. Approximately 95% of this output occurs in developing countries, where it serves as a staple food and an essential source of vitamins and minerals [36]. Remarkably, sweet potato is also among the growing number of naturally transgenic plants: its genome contains ancient insertions of *Agrobacterium*-derived T-DNA (*Ib*T-DNA), the functional significance of which remains poorly understood. The *Ib*T-DNA regions, including *rol*-like genes, exhibit structural conservation across a broad range of cultivated *I. batatas* genotypes, as shown by recent genomic studies [7,9]. Recent studies have also identified similar horizontally acquired T-DNA fragments in other widely consumed plant species, such as tea [37] and persimmon [38]. Based on current estimates, approximately 7% of sequenced dicot species harbor *Agrobacterium*-derived sequences [39], suggesting that thousands of plant species may contain such elements. As these natural transgenes are hypothesized to influence host physiology and environmental adaptability, their characterization may facilitate the development of improved cultivars and inform strategies for enhancing stress resilience. In this study, we employed CRISPR/Cas9-mediated mutagenesis to explore the potential functional roles of two such genes, Ib-rolB/C and Ib-rolD-like, located within IbT-DNA2.

Mutations in *Ib-rolB/C* had minimal impact on callus morphology and biomass accumulation, suggesting that this gene plays a limited role in growth regulation under the tested conditions. In contrast, *Ib-rolD-like* mutants exhibited significant growth impairments, including reduced biomass accumulation and altered morphology. These findings are consistent with the known function of *rolD* in promoting growth through proline metabolism and stress response pathways [40]. In particular, transposon insertions and short deletions in the *rolD* gene of *Agrobacterium rhizogenes* strain A4 were shown to impair the growth of transgenic roots in *Kalanchoë diagremontiana*, compared to roots induced by wild-type *A. rhizogenes* T-DNA [41]. The *Ib-rolD-like* gene similarly exhibits ornithine-dependent NAD^+^ reduction and promotes proline accumulation in transgenic *Arabidopsis*, indicating a conserved biochemical function [8]. Proline is a key regulator of osmotic balance, cell wall expansion, and flowering in plants, and its disruption may explain the observed reductions in growth indices [42]. Proline serves as the precursor for hydroxyproline residues, which are key structural components of hydroxyproline-rich glycoproteins such as extensins and arabinogalactan proteins [43]. These proteins are structural constituents of the plant cell wall and are thought to play essential roles in regulating cell division, wall self-assembly, and extension [44]. The downregulation of *IbCycD3;1*, *IbARF*, and *IbEXP1* in *Ib-rolD-like* mutants further supports its role in cell cycle progression, auxin signaling, and cell wall expansion [45,46,47]. Interestingly, the effects of *Ib-rolD-like* mutations in *I. batatas* were more pronounced than those reported for its *Agrobacterium* homolog. For example, insertion of the *rolD* gene in tomato primarily affected flowering and stress responses without significantly impacting vegetative growth [13].

Mutations in *Ib-rolB/C* led to a reduction in chlorogenic acid derivatives (CQAs), with levels of 3,5-diCQA and other CQAs decreasing by up to 2.5-fold. A similar trend was observed following transient editing of the same gene in leaves, where the total polyphenol content decreased by up to threefold. Although transient agroinfiltration is inherently variable, the observed reduction in total phenolic content in *Ib-rolB/C*-edited leaves was consistent with the metabolic trends seen in callus lines. These findings align with our previous study, in which overexpression of *Ib-rolB/C* under the control of a viral promoter in *I. batatas* callus cultures resulted in a significant increase in phenolic compound accumulation [16]. The observed downregulation of key biosynthetic genes (*IbPAL*, *IbC4H*) in *Ib-rolB/C* mutants further supports the hypothesis that this gene modulates the phenylpropanoid pathway, likely via signaling mechanisms regulating secondary metabolism. In contrast, *Ib-rolD-like* mutations had minimal effects on CQA content and secondary metabolism-related gene expression. This observation aligns with earlier reports indicating that the *rolD* gene from *A. rhizogenes* has relatively modest effects on plant secondary metabolism [10].

Phenolic compounds such as CQAs are known to play critical roles in plant defense, protection against UV radiation, bacterial pathogens, and a broad range of insect herbivores [48,49,50]. Apart from their importance as defense molecules, CQAs also contribute to sweet potato’s nutritional and antioxidant properties. Specifically, CQAs, especially di-CQA and triCQA, exert antioxidant activity primarily via free radical scavenging, metal chelation, and inhibition of lipid peroxidation [51]. Beyond antioxidant functions, CQAs have been shown to modulate the β-catenin/Tcf-4 and Wnt signaling pathways through inhibition of Tcf-4 transcription [52], contribute to neuroprotection [53], and demonstrate antimutagenic activity against heterocyclic amines [54]. These multifunctional roles support their relevance as indicators for evaluating the metabolic effects of *rol* gene disruption in sweet potato, while also leaving room for future studies to explore additional metabolic pathways potentially regulated by the *Ib-rolB/C* and *Ib-rolD-like* genes.

Taken together, this study highlights the utility of CRISPR/Cas9 genome editing for exploring the functions of natural transgenes in sweet potato. Using this approach, we obtained evidence that *Ib-rolB/C* and *Ib-rolD-like* may play distinct roles in sweet potato biology: *Ib-rolB/C* appears to influence secondary metabolism, while *Ib-rolD-like* is potentially involved in growth-related processes. To our knowledge, this is the first report to generate targeted knockouts of naturally transgenic *Agrobacterium*-derived genes in sweet potato, offering new insights into their possible biological relevance. This strategy may also support the broader adoption of CRISPR/Cas9 in functional studies and targeted trait improvement in root and tuber crops, where genome editing holds considerable promise for enhancing food security [55,56,57].

However, two important limitations should be noted. First, our experiments were conducted in callus cultures, which represent dedifferentiated tissue with physiological and transcriptional profiles that differ from those of mature, intact plants. As such, caution is warranted when extrapolating our findings to whole-plant systems. Second, while our results demonstrate functional integration of *Ib-rolB/C* and *Ib-rolD-like* into host regulatory networks, they do not provide evidence of evolutionary advantage. The observed effects may result from epistatic interactions or reflect historical genomic accommodation, rather than representing an adaptive advantage. Future studies involving whole-plant models, population-level analyses, and comparative genomics will be essential to evaluate the broader biological and evolutionary significance of these natural transgenes.

## 4. Materials and Methods

### 4.1. Plant Cell Culture

Callus cultures of *Ipomoea batatas* (sweet potato) were established from surface-sterilized leaf explants collected from 40-day-old plants of the cultivar ‘Taizhong’, grown in well-drained sandy soil (pH 6.0–6.5). For sterilization, leaf segments were immersed in 1% mercuric chloride for 30 s, followed by three rinses with autoclaved distilled water. Callus induction was performed on Murashige and Skoog (MS) medium supplemented with 1 mg/L 2,4-dichlorophenoxyacetic acid (2,4-D). For subsequent proliferation and maintenance, calli were transferred to MS medium containing 0.5 mg/L 4-chlorophenoxyacetic acid (4-CPA), previously identified as the most effective plant growth regulator compared to 2,4-D and 6-benzylaminopurine (BAP) for promoting active cell division and secondary metabolite accumulation [58]. Cultures were incubated in complete darkness at 24 °C and subcultured every 30 days.

### 4.2. sgRNA Design and Plasmid Construction

Two gRNA spacers were designed to target the *Ib-rolB/C* and *Ib-rolD-like* genes within the *I. batatas* cT-DNA2 region (GenBank accession no. KM052617). The target sequences were as follows: gRNA1 (AAGAGCCTCAGAGTCCGGAT; the *Kpn*2I restriction site is underlined) for *Ib-rolB/C* and gRNA2 (ATATTAACCTTAGATGTCGA(C); the *Sal*I restriction site is underlined, with the final cytosine (C) located within the PAM sequence) for *Ib-rolD-like*. Site-directed mutagenesis was carried out using gRNA-specific primers (Supplementary Table S1) and the pUC119-gRNA [59], enabling the incorporation of the designed protospacer sequences into a frame with the gRNA scaffold under the control of the *Arabidopsis thaliana* U6 (AtU6) promoter.

The resulting sgRNA expression cassettes were amplified by polymerase chain reaction (PCR) using primers containing I-*Ceu*I restriction sites (Supplementary Table S1). These cassettes were subsequently ligated into the same restriction sites of the linearized binary vector pPZP-RCS2-NPTII/pcoCas9, previously obtained [60]. This vector contains a plant codon-optimized Cas9 gene (*pcoCas9*) under the control of a double cauliflower mosaic virus (CaMV) 35S promoter, and encodes the neomycin phosphotransferase (*nptII*) gene for kanamycin resistance. The final constructs, pPZP-RCS2-NPTII/pcoCas9/gRNA1 and pPZP-RCS2-NPTII/pcoCas9/gRNA2, were introduced into *Agrobacterium tumefaciens* strain EHA105/pTiBo542 by electroporation using the Gene Pulser (Bio-Rad Laboratories, Hercules, CA, USA), following the manufacturer’s protocol. The transformed *A. tumefaciens* cells were plated on Luria-Bertani (LB) agar containing spectinomycin (300 mg/L), streptomycin (200 mg/L), and rifampicin (150 mg/L), and incubated overnight at 28 °C. Successful transformations were verified by PCR using gene-specific primers.

### 4.3. Transformation of I. batatas Callus Culture

Transgenic callus cultures of *I. batatas* were established via transformation with *A. tumefaciens* carrying either the pPZP-RCS2-NPTII/pcoCas9/gRNA1 or pPZP-RCS2-NPTII/pcoCas9/gRNA2 construct. Transformation was carried out following the method of Vasyutkina et al. [16]. In brief, actively growing callus was co-cultivated with the *Agrobacterium* suspension in liquid MS medium containing 200 µM acetosyringone for 48 h. After co-cultivation, the callus was transferred onto solid MS medium supplemented with 500 mg/L cefotaxime to eliminate bacterial cells and 50 mg/L kanamycin for the selection of transformed cells.

To optimize CRISPR/Cas9-mediated editing, we systematically evaluated three transformation parameters: the *A. tumefaciens* strain (AGL0, EHA105, and GV3101), the optical density of the bacterial suspension (OD_600_ = 0.1, 0.5, and 1.0), and the developmental stage of the callus tissue (two-week-old and four-week-old cultures). For comparative assessment, each condition was tested in at least three independent biological replicates. To estimate the relative frequency of induced mutations, a portion of the transformed callus biomass was harvested 14 days post-transformation and total genomic DNA was extracted.

For the generation of stable lines, transformed calli were maintained on selective medium for three months with subculturing every four weeks. Several kanamycin-resistant cell lines were selected from independently transformed callus aggregates. Three transgenic cell lines for each target (*Ib-rolB/C* and *Ib-rolD-like*) were chosen for further experiments. All subsequent phenotypic and molecular analyses were conducted after these mutant lines had undergone extended growth in the absence of antibiotic selection. T-DNA integration in these selected transgenic lines was confirmed via PCR analysis using primers specific to the *nptII* gene (Supplementary Table S1). To exclude the possibility of residual Agrobacterium contamination, an additional PCR was performed using primers targeting the *virD2* gene, which is located outside the T-DNA region on the Agrobacterium *A. tumefaciens* plasmid. All tested callus lines yielded positive amplification for *nptII* and negative results for *virD2*, confirming successful T-DNA integration and the absence of bacterial cells (Supplementary Figure S2). Transgenic and untransformed (control) callus cultures were subsequently cultivated under identical conditions for comparative analyses.

### 4.4. Transient Transformation of I. batatas Leaves

Two agroinfiltration methods were employed to transform *I. batatas* leaves: syringe agroinfiltration and vacuum agroinfiltration. For syringe agroinfiltration, *A. tumefaciens* cells carrying the pPZP-RCS2-NPTII/pcoCas9/gRNA1 or pPZP-RCS2-NPTII/pcoCas9/gRNA2 plasmids were introduced into sweet potato leaves using a syringe without a needle. Infiltration was performed on the abaxial (lower) surface of apical young leaves, 2–3 days post-emergence. After infiltration, the plants were maintained in a greenhouse under controlled conditions for 4 days to facilitate transient expression of the transgene. For vacuum agroinfiltration, detached leaves of a similar developmental stage were exposed to infiltration using an Eppendorf Concentrator Plus, as previously described [61]. The transformed leaves were then incubated in a climate chamber for 4 days. In both methods, leaves infiltrated with *A. tumefaciens* EHA105 carrying the empty vector pPZP-RCS2-NPTII/pcoCas9 served as controls.

### 4.5. Genotyping and Sequencing of the Gene Mutations

Genomic DNA was extracted from transgenic *I. batatas* callus cultures using the cetyl trimethylammonium bromide (CTAB) method. Target regions containing the CRISPR/Cas9-induced mutations were amplified using gene-specific primer pairs, rolB/C-target and rolD-like-target (Supplementary Table S1), producing fragments of 668 bp and 552 bp for *Ib-rolB/C* and the *Ib-rolD-like*, respectively.

Purified PCR products were subjected to restriction enzyme digestion with *Kpn*2I (for *Ib-rolB/C*) and *Sal*I (for *Ib-rolD-like*). To confirm the presence of CRISPR/Cas9-induced mutations, DNA fragments resistant to restriction enzyme digestion were purified, ligated into the pJET1.2/blunt vector (Thermo Fisher Scientific Inc., Waltham, MA, USA), and sequenced using the ABI 3500 Genetic Analyzer (Applied Biosystems, Foster City, CA, USA). Sequence alignments were performed against the reference genes *Ib-rolB/C* and *Ib-rolD-like* from *I. batatas* genome to identify insertions, deletions, or other modifications at the target sites.

Mutation rates were determined using the protocol described by Shan et al. [62]. The digested products were resolved on an agarose gel, and band intensities were quantified using GelAnalyzer 23.1.1 (available at www.gelanalyzer.com (accessed on 31 January 2025)) by Istvan Lazar Jr., PhD and Istvan Lazar Sr., PhD, CSc. The total intensities were calculated by summing the individual band intensities, subtracting background fluorescence. Mutation frequency was estimated as the ratio of the intensity of the undigested (uncut) band to the total band intensity.

### 4.6. Gene Expression Analysis

Total RNA was isolated from 150 mg of *I. batatas* callus tissue using the Lira reagent kit (Biolabmix, Novosibirsk, Russia), following the manufacturer’s protocol. First-strand complementary DNA (cDNA) was synthesized, and quantitative real-time PCR (qPCR) analysis was performed as previously described by Balabanova et al. [63]. All reactions were conducted on a CFX96 thermal cycler (Bio-Rad Laboratories, Hercules, CA, USA) using 2 × BioMaster HS-qPCR SYBR Blue (Biolabmix, Novosibirsk Oblast, Russia). Gene-specific primer pairs used in the qPCR experiments are detailed in Supplementary Table S1. The ubiquitin (*UBQ*) gene of *I. batatas* served as the reference gene for normalization. *UBQ* was selected based on a comparative stability analysis against other commonly used reference genes, including elongation factor-1 alpha (*EF-1α*), glyceraldehyde-3-phosphate dehydrogenase (*GAPDH*), actin (*ACT*), and tubulin (*TUB*). Expression stability was evaluated using the RefFinder algorithm [64], which integrates multiple statistical approaches. Supplementary Figure S3 shows the relative expression stability rankings of the candidate reference genes. Primer efficiencies exceeding 95% were confirmed by generating standard curves from serial dilutions of purified PCR products. Each expression analysis was performed in triplicate, with three independent biological replicates derived from separate RNA extractions, and three technical replicates for each biological sample. Relative expression levels of the target genes were quantified using the −ΔΔCt method, with calculations performed in CFX Manager Software ver. 3.1 (Bio-Rad Laboratories, USA).

### 4.7. Estimation of Secondary Metabolite Content

For secondary metabolite analysis, *I. batatas* callus tissue was dried, ground into a fine powder, and extracted with two volumes of 80% methanol. The extracts were analyzed using a 1260 Infinity analytical high-performance liquid chromatography (HPLC) system (Agilent Technologies, Santa Clara, CA, USA), equipped with a photodiode array detector (DAD), at the Instrumental Centre of Biotechnology and Gene Engineering of FSC Biodiversity FEB RAS.

Separation was performed on a Zorbax C18 column (150 mm × 2.1 mm i.d., 3.5 μm particle size, Agilent Technologies, Santa Clara, CA, USA). The mobile phase consisted of 0.1% formic acid in water (A) and acetonitrile (B), applied in a linear gradient from 5% to 100% B over 30 min, held for 5 min, with a flow rate of 0.2 mL/min. UV spectra were recorded with a DAD in the range between 200 and 400 nm.

The HPLC system was interfaced with a Bruker HCT Ultra PTM Discovery ion trap mass spectrometer (Bruker Daltonik GmbH, Bremen, Germany) equipped with an electrospray ionization (ESI) source. The MS analysis was conducted in negative ion mode with the following parameters: *m*/*z* range 100–1000; drying gas (N_2_) flow rate 8.0 L/min; nebulizer pressure 25 psi; capillary voltage 3.8 kV; drying gas temperature 325 °C. Tandem mass spectra were acquired in Auto-MS^2^ mode with collision energy ramping and a fragmentation amplitude of 1 V.

Quinic acid derivatives were quantified by HPLC-DAD at 325 nm, based on four-point external calibration curves constructed using reference standards, chlorogenic acid and 1,3-diCQA (cynarin), obtained from Sigma-Aldrich (St. Louis, MO, USA). The procedure followed the detailed protocol described by Vasyutkina et al. [16]. A representative chromatographic profile of a methanolic extract from *I. batatas* callus, recorded by HPLC and ESI-MS, is shown in Supplementary Figure S1.

### 4.8. Statistical Analysis

All data were expressed as mean ± standard error (SE). Statistical evaluation of differences between two independent groups was performed using Student’s *t*-test. For comparisons involving multiple groups, analysis of variance (ANOVA) was conducted, followed by Fisher’s protected least significant difference (PLSD) post hoc test for inter-group comparisons. A significance level of *p* < 0.05 was considered statistically significant.

## 5. Conclusions

In this study, we applied CRISPR/Cas9-mediated mutagenesis to investigate the potential functional roles of the *Ib-rolB/C* and *Ib-rolD-like* genes in the naturally transgenic plant *Ipomoea batatas*. Optimization of the transformation protocol enabled efficient genome editing in sweet potato callus cultures and may serve as a basis for future functional studies in this system. Mutations in *Ib-rolB/C* did not affect callus morphology or growth but were associated with a consistent decrease in chlorogenic acid derivatives, supporting its possible role in the regulation of phenylpropanoid metabolism. Transient *in planta* editing of *Ib-rolB/C* produced similar effects, with reduced total polyphenol content. In contrast, *Ib-rolD-like* mutants displayed lower biomass accumulation and downregulation of cell cycle–related genes, suggesting a possible link to growth regulation, potentially via proline metabolism. These findings suggest that *Ib-rolB/C* and *Ib-rolD-like* may contribute differentially to secondary metabolism and cell growth in sweet potato, although further studies in whole-plant systems are needed to fully confirm these roles.

## Figures and Tables

**Table 1:** Content of CQAs in control and transgenic *Ipomoea batatas* callus lines with CRISPR/Cas9-induced mutations in *Ib-rolB/C* and *Ib-rolD-like* genes (mg/g dry weight).

Metabolites	Control	*Ib-rolB/C* Mutants			*Ib-rolD-like* Mutants		
		BC1	BC2	BC3	DL1	DL2	DL3
CGA	0.74 ± 0.05 ^A^	0.61 ± 0.05 ^B^	0.73 ± 0.07 ^A^	0.64 ± 0.05 ^B^	0.91 ± 0.12 ^B^	0.79 ± 0.010 ^A^	0.78 ± 0.09 ^A^
3,4-diCQA	0.02 ± 0.01 ^B^	0.02 ± 0.01 ^B^	0.02 ± 0.01 ^B^	0.02 ± 0.01 ^B^	0.04 ± 0.01 ^A^	0.02 ± 0.01 ^B^	0.02 ± 0.01 ^B^
3,5-diCQA	4.72 ± 0.29 ^A^	3.01 ± 0.20 ^B^	3.61 ± 0.25 ^B^	3.15 ± 0.18 ^B^	4.02 ± 0.50 ^A^	4.25 ± 0.49 ^A^	4.24 ± 0.38 ^A^
4,5-diCQA	0.07 ± 0.01 ^A^	0.03 ± 0.02 ^B^	0.04 ± 0.02 ^B^	0.03 ± 0.02 ^B^	0.04 ± 0.01 ^B^	0.04 ± 0.01 ^B^	0.04 ± 0.01 ^B^
3C-5CoQA	0.06 ± 0.01 ^A^	0.02 ± 0.02 ^B^	0.02 ± 0.02 ^B^	0.03 ± 0.02 ^B^	0.03 ± 0.01 ^B^	0.05 ± 0.01 ^A^	0.05 ± 0.01 ^A^
3F-5CQA	0.24 ± 0.02 ^A^	0.20 ± 0.01 ^A^	0.11 ± 0.01 ^B^	0.10 ± 0.02 ^B^	0.26 ± 0.02 ^A^	0.22 ± 0.02 ^A^	0.22 ± 0.01 ^A^
3C-5FQA	0.20 ± 0.02 ^A^	0.02 ± 0.01 ^B^	0.09 ± 0.01 ^B^	0.08 ± 0.01 ^B^	0.12 ± 0.02 ^B^	0.17 ± 0.02 ^A^	0.17 ± 0.02 ^A^
Total CQAs	6.06 ± 0.51 ^A^	3.91 ± 0.31 ^B^	4.62 ± 0.33 ^B^	4.04 ± 0.32 ^B^	5.43 ± 0.68 ^A^	5.53 ± 0.75 ^A^	5.52 ± 0.69 ^A^

Different superscript letters indicate statistically significant differences in means (*p* < 0.05), Fisher’s LSD.

## Data Availability

The original contributions presented in this study are included in the article/Supplementary Material. Further inquiries can be directed to the corresponding authors.

## Supplementary Materials

The following supporting information can be downloaded at: https://www.mdpi.com/article/10.3390/plants14243708/s1, Table S1: List of primer sequences used in this study. Figure S1: Representative chromatographic profile of methanolic extract from *I. batatas* callus recorded by HPLC with UV detection (λ = 325 nm), coupled to ESI-MS in negative ion mode. Figure S2: PCR verification of T-DNA integration and absence of residual *A. tumefaciens* in transgenic *I. batatas* callus lines. Figure S3: Expression stability analysis of candidate reference genes in *I. batatas* callus cultures as calculated by RefFinder.
