# Supplementary material for: Functional Analysis of Naturally Integrated Rol Genes in Sweet Potato via CRISPR/Cas9 Genome Editing
Source: Plants (Basel). 2025 Dec 5;14(24):3708. doi: 10.3390/plants14243708 (PMC12737136; doi:10.3390/plants14243708)
Supplement: Supplementary file 1 [file plants-14-03708-s001.zip › Supplementary Figures.pdf]

## Supplementary Materials

# Functional Analysis of Naturally Integrated *Rol* Genes in Sweet Potato via CRISPR/Cas9 Genome Editing


**Yury Shkryl *, Yulia Yaroshenko, Valeria Grigorchuk, Victor Bulgakov and Yulia Yugay ***

Federal Scientific Center of the East Asia Terrestrial Biodiversity of the Far East Branch of Russian Academy of Sciences, Vladivostok, 690022, Russia

* Correspondence: author: Yury Shkryl and Yulia Yugay, Federal Scientific Center of the East Asia Terrestrial Biodiversity of the Far East Branch of Russian Academy of Sciences, 159 Stoletija Str., Vladivostok, 690022, Russia; Tel: 007 4232 312129; Fax: 007 4232 310193; E-mail address: yn80@mail.ru, yuya1992@mail.ru


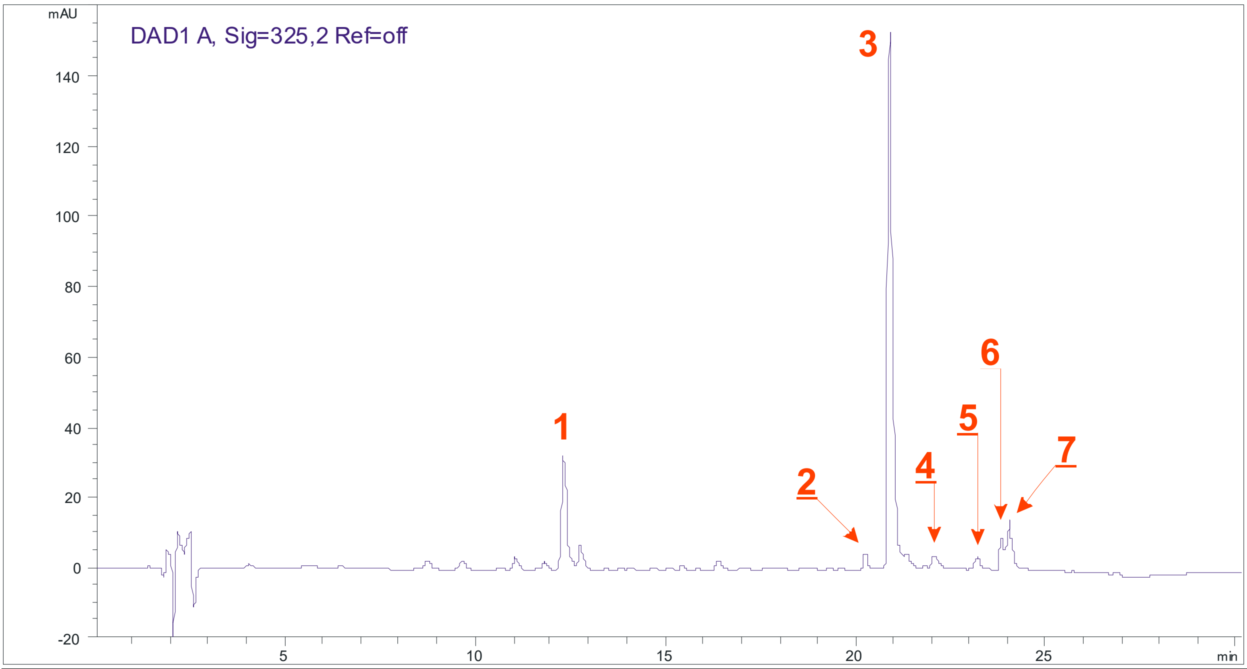


**Peak 1.**
RT = 12.3 min,
UVmax at 323 nm

[M-H]$^-$ at *m/z* 353

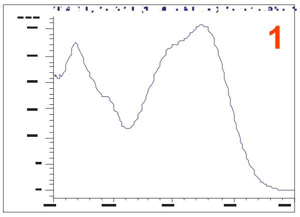


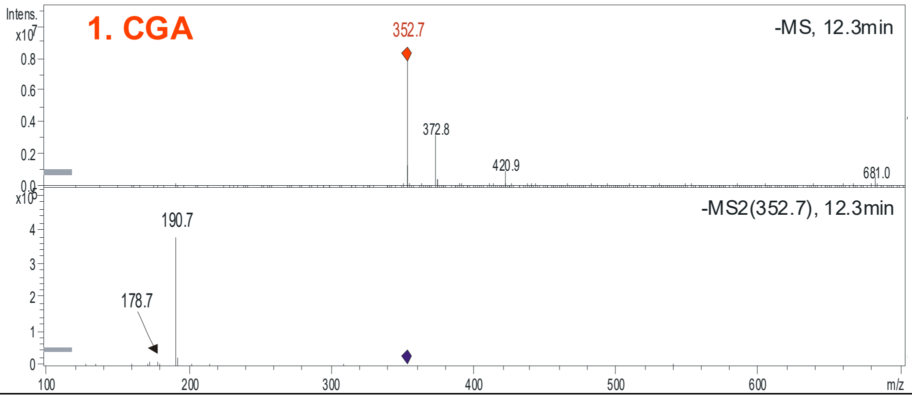


**Peak 2.**
RT = 20.3 min,
UVmax at 323 nm

[M-H]- at *m/z* 515

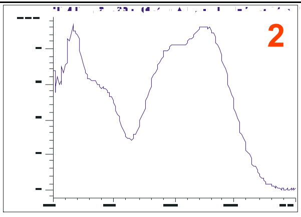


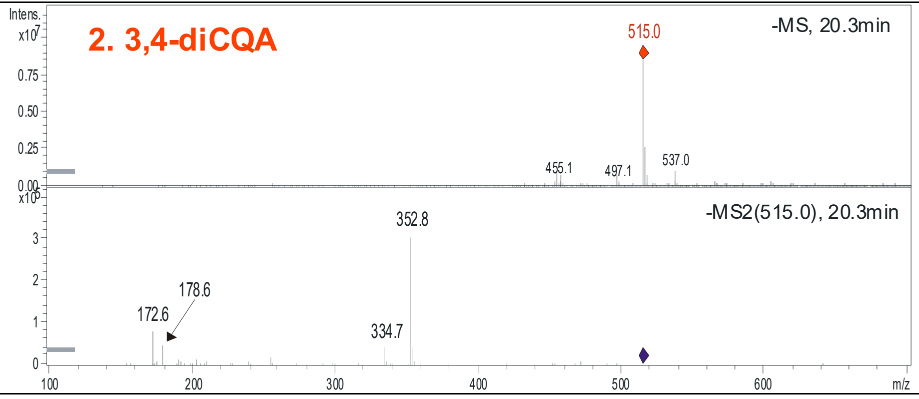


**Peak 3.**
RT = 20.9 min,
UVmax at 326 nm

[M-H]- at *m/z* 515

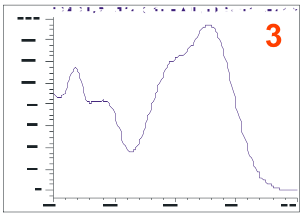


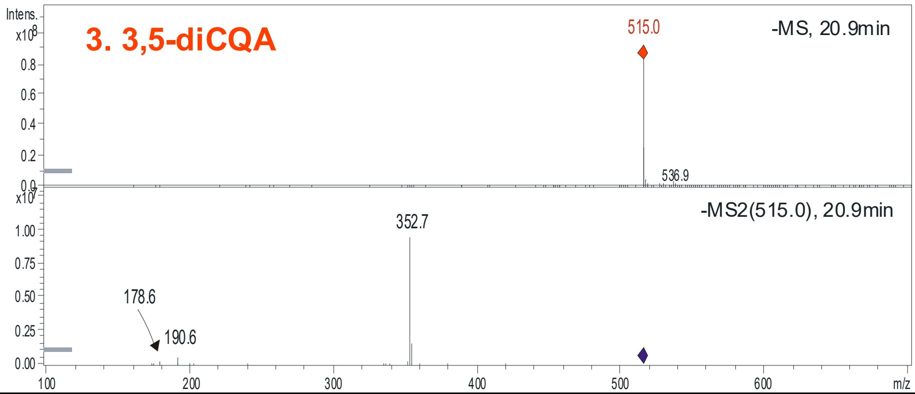


**Peak 4.**
RT = 22.1 min,
UVmax at 326 nm

[M-H]- at *m/z* 515

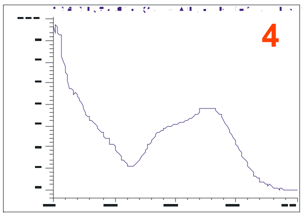


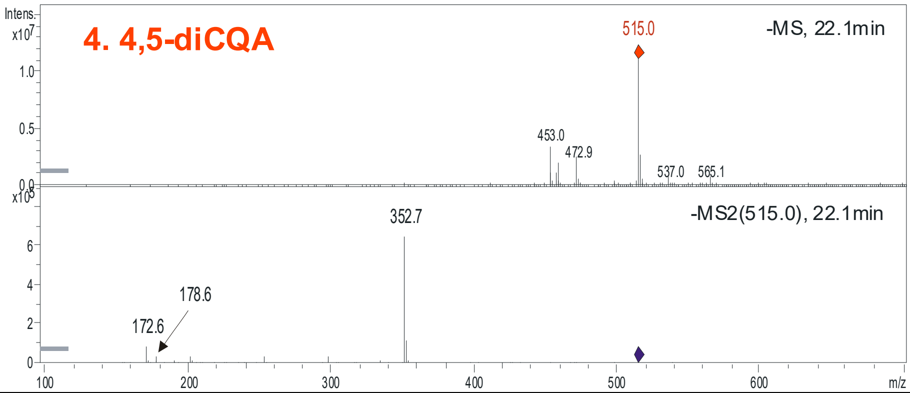


**Peak 5.**
RT = 23.2 min,
UVmax at 318 nm

[M-H]- at *m/z* 499

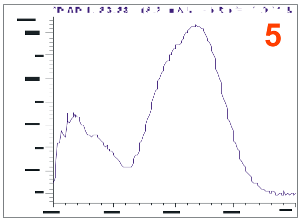


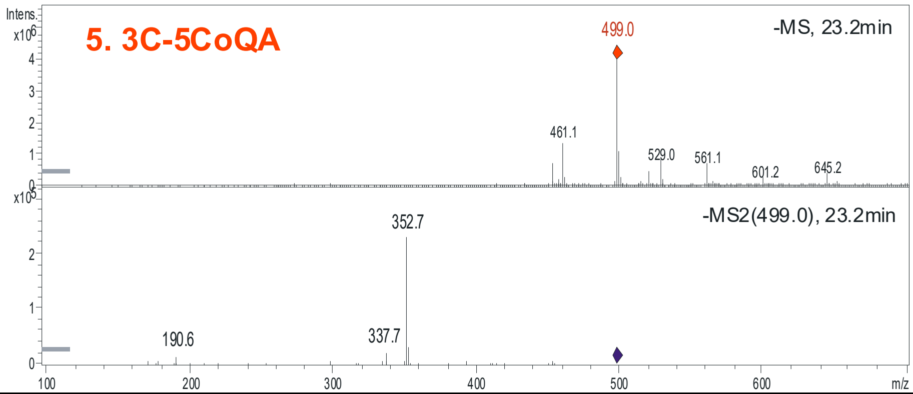


**Peak 6.**
RT = 23.8 min,
UVmax at 326 nm

[M-H]- at *m/z* 529

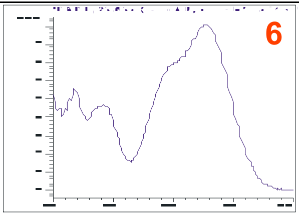


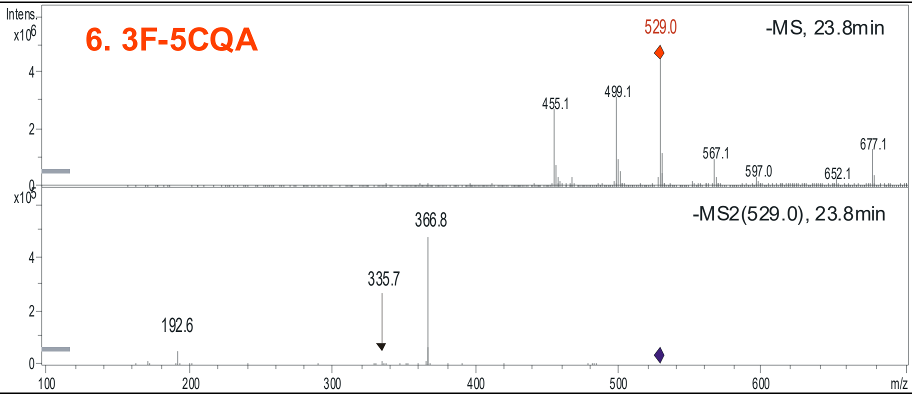


**Peak 7.** [M-H]- at *m/z* 529
RT = 24.1 min,
UVmax at 326 nm

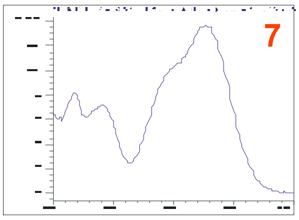


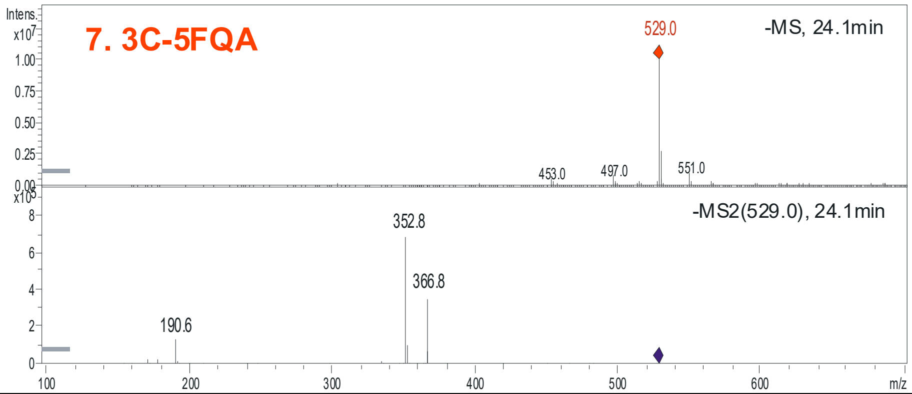


**Figure S1.** Representative chromatographic profile of methanolic extract from *I. batatas* callus recorded by HPLC with UV detection ($\lambda$ = 325 nm), coupled to ESI-MS in negative ion mode. Seven major peaks were identified as quinic acid derivatives based on retention time (RT), UV absorption maxima ($UV_{max}$), and deprotonated molecular ions $[M–H]^-$. Peak 1 (RT = 12.3 min, *m/z* 353) corresponds to chlorogenic acid (CGA). Peaks 2–7 (RT = 20.3–24.1 min, m/z 499–529) correspond to various diacyl derivatives, namely: *3,4-di-O-caffeoylquinic acid* (3,4-diCQA), *4,5-di-O-caffeoylquinic acid* (4,5-diCQA), *3-O-caffeoyl-5-O-coumaroylquinic acid* (3C-5CoQA), *3-O-feruloyl-5-O-caffeoylquinic acid* (3F-5CQA), and *3-O-caffeoyl-5-O-feruloylquinic acid* (3C-5FQA).

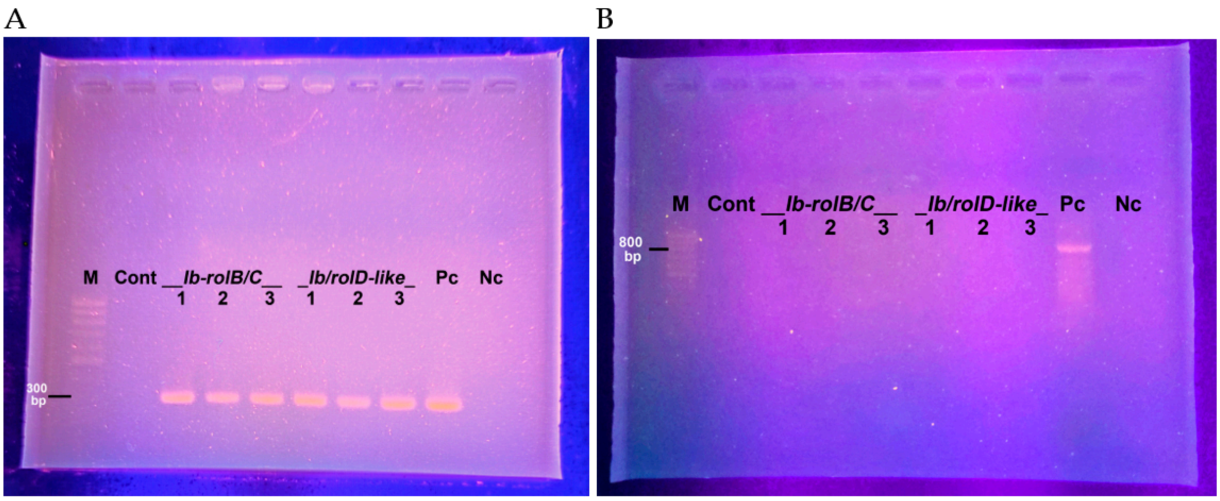


**Figure S2.** PCR verification of T-DNA integration and absence of residual *A. tumefaciens* in transgenic *I. batatas* callus lines. (A) Amplification of the *nptII* gene confirms the presence of the T-DNA construct in all tested transgenic lines. (B) No amplification of the *virD2* gene was observed, indicating the absence of bacterial contamination. Cont – wild-type (non-transformed) callus line; Pc – positive control (plasmid DNA); Nc – negative control (water); M – 1 kb DNA ladder.

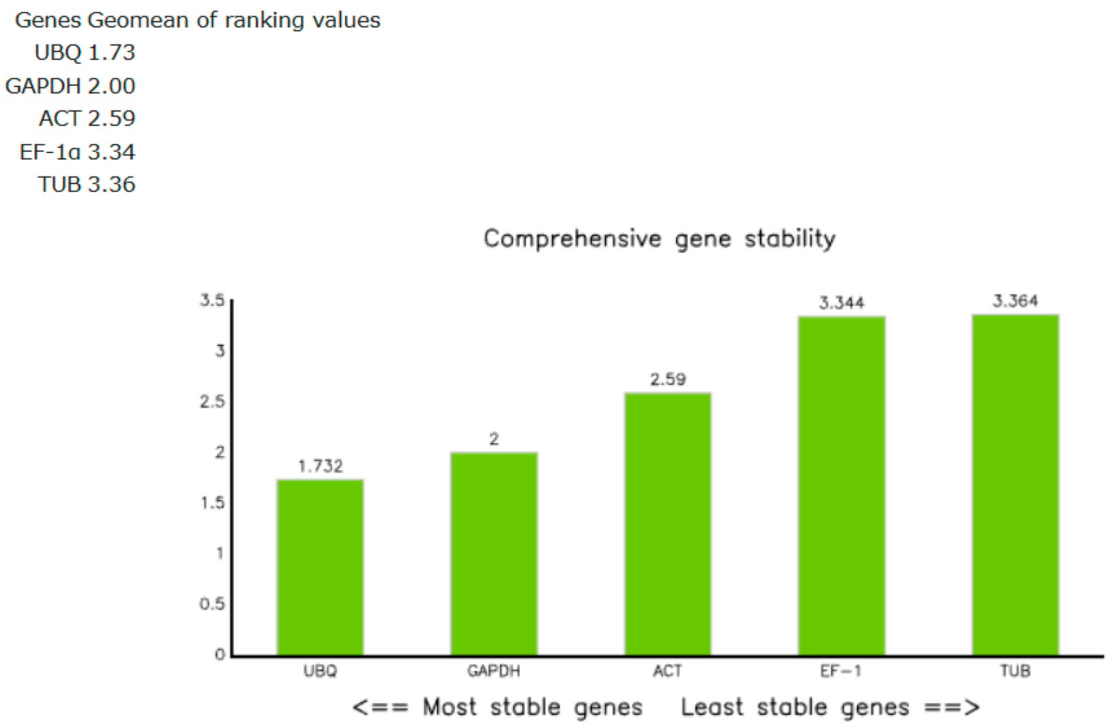


**Figure S3.** Expression stability analysis of candidate reference genes in *I. batatas* callus cultures as calculated by RefFinder. The bar graph shows the geometric mean of ranking values across four algorithms (GeNorm, NormFinder, BestKeeper, and ΔCt method), with lower values indicating greater stability. Gene abbreviations: *UBQ*, ubiquitin; *GAPDH*, glyceraldehyde-3-phosphate dehydrogenase; *ACT*, actin; *EF-1α*, elongation factor 1-alpha; *TUB*, tubulin.
