# Supplementary material for: Functional Analysis of Naturally Integrated Rol Genes in Sweet Potato via CRISPR/Cas9 Genome Editing
Source: Plants (Basel). 2025 Dec 5;14(24):3708. doi: 10.3390/plants14243708 (PMC12737136; doi:10.3390/plants14243708)
Supplement: Supplementary file 1 [file plants-14-03708-s001.zip › Supplementary Table.pdf]

Supplementary Materials

Functional Analysis of Naturally Integrated *Rol* Genes in Sweet Potato via CRISPR/Cas9 Genome Editing

Yury Shkryl *, Yulia Yaroshenko, Valeria Grigorchuk, Victor Bulgakov and Yulia Yugay *

Federal Scientific Center of the East Asia Terrestrial Biodiversity of the Far East Branch of Russian Academy of Sciences, Vladivostok, 690022, Russia

* Correspondence: author: Yury Shkryl and Yulia Yugay, Federal Scientific Center of the East Asia Terrestrial Biodiversity of the Far East Branch of Russian Academy of Sciences, 159 Stoletija Str., Vladivostok, 690022, Russia; Tel: 007 4232 312129; Fax: 007 4232 310193; E-mail address: yn80@mail.ru, yuya1992@mail.ru

Table S1. List of primer sequences used in this study.

Primer name (GenBank accession no.)	Forward (5′ to 3′)	Reverse (5′ to 3′)
Construction of sgRNA expression cassettes		
gRNA1	GAAGAGCCTCAGAGTCCGGATGT TTTAGAGCTAGAAATAGC	ATCCGGACTCTGAGGCTCTTCAATCACT ACTTCGTCTCTAAC
gRNA2	GATATTAACCTTAGATGTCGAGTT TTAGAGCTAGAAATAGC	TCGACATCTAAGGTTAATATCAATCACT ACTTCGTCTCTAAC
pUC119-*Ceu*I	CATTCGCTACCTTAGGACCGTTAT AGTTAGAGCTCAGAAATCTCAAA ATTC	ATGTAACTATAACGGTCCTAAGGTAGCG AGTACAAGAAAGCTGGGTCTAG
Mutant screening and sequencing		
rolB/C-target (KM052617)	GTATGCGCGAGCGGTTTTGC	GCCACAAGCCAATACATCATA
rolD-like-target (KM052617)	GATCCCATGCATAGCACCAT	TGAAAACCTTTATGTCACGCTT
qPCR analysis		
*npt*II (AY818371)	TCGCGCCAGCCGAACTGTTC	TCAGAAGAACTCGTCAAGAAG
virD2 (CP067035)	GCCGAGATGTTTGGGTCAGG	GCTGTCATCGCGACCGTCGG
IbPAL (MN823653, D78640)	ATGCTGACCGGAGAGAAGG	AACAGATAGGAAGAGGAGCC
IbC4H (GQ373157)	GAGTTCCGCCCAGAGAGG	CAAGATGTGGAGACTGAACT
Ib4CL (AB469557)	GTCCCTGTTGCGTTTGTGGT	TTATTGCGGCGTGGAAGGTG
IbHCT (AB576768)	GACGGAGGATCAAGAAACCA	GGATCGCAGGTAATCGTTGT
IbHQT (AB576769)	CACAGTCTACATTCTCCCCA	TCAGAAATCATACAAGAACTCC
IbEXP1 (DQ515800)	GATCAAGAACCGGGTGGCA	AGACGACGCTGCGGCTGT

IbNAC1 (MG657368)	TACATGCACTTCGACACGTC	AAGGTTTGTGCATGTATGTGAA
IbARF (JX177359)	GGGATGCTGTGTTGCTTGTG	TGACGTTGACGGAGAGAGTG
IbCycD3;1 (AB478416)	TGAGCCCTGCAATGCCATG	CATTGGAGCTATCAGAGGTG
IbUBQ (JX177358)	AGTCCACTCTCCACCTCGTC	CCTTTCCAGACTCATCCACC
IbACT (EU250003)	GTTATGGTTGGGATGGGACA	GTTGTAGAAAGTGTGATGCCAG
IbEF1α (HX977465)	CTCCAAGGATGACCCAGC	GGCAGTCGAGAACAGGAG
IbTUB (BM878762)	TCCAAACCAACCTTGTACCC	TTTTGCCATCATGCTTGAGG
IbGAPDH (JX177362)	CGCTCACTTGAAGGCTGGT	AGGAGCAAGGCAGTTGGTAG
